# DPM as a radiation transport engine for PRIMO

**DOI:** 10.1186/s13014-018-1188-6

**Published:** 2018-12-27

**Authors:** Miguel Rodriguez, Josep Sempau, Christian Bäumer, Beate Timmermann, Lorenzo Brualla

**Affiliations:** 1 Centro Médico Paitilla, Calle 53 y ave. Balboa, Panama City, Panama; 2 Instituto de Investigaciones Científicas y de Alta Tecnología, INDICASAT-AIP, City of Knowledge, Building 219, Panama City, Panama; 3 Universitat Politècnica de Catalunya, Diagonal 647, Barcelona, E-08028 Spain; 4 West German Proton Therapy Centre Essen (WPE), Hufelandstraße 55, Essen, D-45147 Germany; 5 West German Cancer Center (WTZ), Hufelandstraße 55, Essen, D-45147 Germany; 6 University Hospital Essen, Hufelandstraße 55, Essen, D-45147 Germany; 7 German Cancer Consortium (DKTK), Hufelandstraße 55, Essen, D-45147 Germany; 8 Department of Particle Therapy, University Hospital Essen, Hufelandstraße 55, Essen, D-45147 Germany

**Keywords:** Monte Carlo, Radiation transport, Linear accelerator

## Abstract

**Background:**

PRIMO is a dose verification system based on the general-purpose Monte Carlo radiation transport code penelope, which implements an accurate physics model of the interaction cross sections and the radiation transport process but with low computational efficiency as compared with fast Monte Carlo codes. One of these fast Monte Carlo codes is the Dose Planning Method (DPM). The purpose of this work is to describe the adaptation of DPM as an alternative PRIMO computation engine, to validate its performance against penelope and to validate it for some specific cases.

**Methods:**

DPM was parallelized and modified to perform radiation transport in quadric geometries, which are used to describe linacs, thus allowing the simulation of dynamic treatments. To benchmark the new code versus penelope, both in terms of accuracy of results and simulation time, several tests were performed, namely, irradiation of a multi-layer phantom, irradiation of a water phantom using a collimating pattern defined by the multileaf collimator (MLC), and four clinical cases. The gamma index, with passing criteria of 1 mm/1%, was used to compare the absorbed dose distributions. Clinical cases were compared using a 3-D gamma analysis.

**Results:**

The percentage of voxels passing the gamma criteria always exceeded 99% for the phantom cases, with the exception of the transport through air, for which dose differences between DPM and penelope were as large as 24%. The corresponding percentage for the clinical cases was larger than 99%. The speedup factor between DPM and penelope ranged from 2.5 ×, for the simulation of the radiation transport through a MLC and the subsequent dose estimation in a water phantom, up to 11.8 × for a lung treatment. A further increase of the computational speed, up to 25 ×, can be obtained in the clinical cases when a voxel size of (2.5 mm)^3^ is used.

**Conclusions:**

DPM has been incorporated as an efficient and accurate Monte Carlo engine for dose estimation in PRIMO. It allows the concatenated simulation of the patient-dependent part of the linac and the patient geometry in static and dynamic treatments. The discrepancy observed between DPM and penelope, which is due to an artifact of the cross section interpolation algorithm for low energy electrons in air, does not affect the results in other materials.

## Background

PRIMO [1, 2] is a computer software that simulates clinical linear accelerators (linacs) and estimates absorbed dose distributions in phantoms and computerized tomography (CT) studies. It combines a graphical user interface with the general-purpose radiation transport Monte Carlo code PENELOPE (version 2011) [3]. It is freely distributed through the website https://www.primoproject.net since 2013.

PENELOPE implements an accurate physics model of the interaction cross sections and the radiation transport process but exhibits a relatively low computational performance compared with fast Monte Carlo codes specifically designed for radiotherapy problems [4]. One such code is the Dose Planning Method (DPM v1.1) [5] which simulates absorbed dose distributions deposited by electron-photon showers in external beam radiotherapy treatments. The open-source code is freely distributed through http://www.upc.es/inte/downloads. The present work describes the adaptation of DPM, hereafter identified as *p*DPM, to the PRIMO system and its subsequent validation.

*p*DPM includes a mixed-geometry model that allows the simulation in voxelized and quadric surface geometries. This capability allows the joined simulation of the linac patient-dependent part and the patient, hence making possible the simulation of dynamic treatments. The scope of including *p*DPM as a simulation engine of PRIMO is to facilitate usage of the latter as a Monte Carlo dose verification system for the routine clinical practice.

## Methods

The guidelines for reporting Monte Carlo simulations, provided by the AAPM Task Group 268 [6], have been followed in the preparation of this work.

### Dose planning method

DPM gains in computing performance derive from various enhancements to the description of particle transport and of the underlying physics models. More precisely, the main features that explain its accuracy and computational efficiency are the following:

- It uses simplified cross section models that are accurate for the energy range typically employed in conventional radiotherapy and for low atomic numbers, such as those encountered inside the patient body. For example, the Klein-Nishina differential cross section [7] is used to describe photon incoherent (Compton) scattering, thus neglecting Doppler broadening and binding effects, which are non-negligible for high *Z* elements or low energies. Similarly, the Møller differential cross section [8] is used to describe electron inelastic collisions with atomic electrons, thus assuming that the target particle is free and at rest. This, again, is valid for low atomic numbers and high energies.
- Photon transport is simulated detailedly using the delta scattering, or Woodcock tracking technique [9], which completely avoids the need to consider intersections with voxel walls.
- For electrons, DPM employs the standard condensed history model, falling into what has been called a mixed scheme for the treatment of energy losses by Berger [10]. It treats large energy transfer collisions detailedly and uses the continuous slowing down approximation to describe the effect of small energy loss interactions. For condensing angular deflections, the code is based on a refinement of the Kawrakow and Bielajew [11] formulation of the Lewis multiple-scattering theory [12], which allows fast random sampling of the scattering angle. The algorithm further relies on the small angle approximation, under which all materials can be characterized by means of a single scattering angle distribution.

The DPM code has been extensively benchmarked and validated by a group from the University of Michigan [13, 14]. It should be noticed that the bulk of the DPM development effort was focused on the electron transport algorithm. There is still room for improvement regarding the application of variance-reduction techniques for photon transport. Despite this fact, the code has been shown to reproduce dose distributions estimated with high-accuracy general-purpose Monte Carlo codes within an error of the order of 1.5% of the maximum dose with a significant increase in computational efficiency [15].

DPM has been employed as a dose distribution calculation engine by other authors. For example, version 3 beta of the ADAC Pinnacle treatment planning system was based on a C++ port of DPM. ADAC was subsequently acquired by Philips Medical Systems in 2000 but the Pinnacle version based on DPM was never released [4]. The code was also integrated into the University of Michigan’s in-house treatment planning system (UMPlan) [15]. Additionally, a prototype of a new treatment planning system based on DPM was also developed by Técnicas Radiofísicas (Zaragoza, Spain) [16].

Some researchers have devoted efforts to further accelerate the code. Thus, for instance, Tyagy and coworkers [17] used the Message Passing Interface (MPI) library to parallelize the algorithm, Weng et al. [18] aimed at vectorizing the code and Jia et al. [19] adapted it to the graphics processing unit (GPU) architecture.

### DPM improvements

#### Parallelization of DPM

One of the limitations of DPM is its lack of support for phase-space files or other sources of particles needed for linac simulation. Furthermore, its sequential code cannot fully exploit the capabilities of parallel processors. These capabilities have been added to *p*DPM as explained in a previous work [20].

#### Mixed geometry model

The developed mixed geometry model combines bodies defined by quadric surfaces and voxels. The aim is to merge the patient-dependent region of the linac, which is modeled by quadrics, and the patient, represented by the voxelized geometry. Therefore, in simulations of dynamic treatments, the transport through both regions can be performed in a single simulation step.

In the mixed model the patient dependent region of the linac is defined according to the rules of PENGEOM, the PENELOPE geometry package, while the voxelized geometry uses the model currently implemented in DPM. To combine both models we rely on an approach that has been used before by Sempau and collaborators in the PENEASY code [2]. Transport in the voxelized geometry proceeds as in the original version of DPM [21] while in the quadric geometry it is performed using the routines included in PENELOPE.

#### Dynamic geometry

Dynamic geometry uses our mixed geometry model to simulate dynamic irradiations, thus allowing changing the positions of multileaf collimators, jaws, gantry, collimator and couch at execution time. To this purpose the simulation is divided into control points, each one defined by a fixed configuration of the aforementioned movable elements. The fraction of the total number of histories that is simulated for each control point equals the fraction of monitor units as specified in the cumulative meterset weight of the DICOM-RTPLAN file.

#### Variance-reduction techniques

Two variance-reduction techniques [22] were implemented in *p*DPM, namely simple particle splitting in the patient and range-rejection of electrons in the internal regions of the MLC and the jaws. Range rejection was implemented through the movable-skins technique [23].

### *p*DPM benchmarks

Simulations presented in this article considered a 6 MV beam of a Clinac-iX linear accelerator equipped with a Varian Millennium 120 MLC. The particle source employed was a phase-space file (PSF) tallied from the simulation of the patient-independent part of the linac using PENELOPE with initial beam parameters *E*=6.2 MeV, FWHM_*E*_=0.186 MeV, FWHM_focal spot size_=0.15 cm and a beam divergence of 2.5 degrees. The PSF produces a dose distribution in water that reproduces well the measured dose profiles.

The assessment of the agreement between dose distributions was done using gamma analysis. The reference data sets were those obtained with PENELOPE while the evaluated data sets were those obtained with *p*DPM. Local gamma analysis was performed with a search volume established according to the distance to agreement (DTA) criterion. The maximum search distance from the reference point to the volume border is calculated as 1.2 *DTA*. Therefore, any evaluated dose point outside the local volume cannot pass the gamma analysis as it would not comply with the DTA criterion. The search step inside the local volume is set such that at least 5 points are sampled in each spatial direction inside the volume and it is required to be at least half the minimum spatial resolution of both dose distributions. Dose sampling inside the local volume is made by tri-linear interpolation. Reference dose values less than 1% of the maximum dose or with uncertainties (2 *σ*) larger than 10% were not included in the analysis. Gamma pass rate (*Γ*_*d*,*DTA*_), i.e. the fraction of points passing gamma analysis with a dose difference *d* (in %) and distance *DTA* (in mm) criteria was evaluated in all cases. For clinical cases, *Γ*_1,1_, *Γ*_2,1_ and *Γ*_2,2_ were evaluated in the region inside the patient’s body, in planning target volumes (PTVs) and in selected organs-at-risk (OARs).

Additionally, the method proposed by Kawrakow and Fippel [24] was used to compare the dose distributions estimated with PENELOPE and pDPM. This method allows to discern systematic differences from those resulting from statistical fluctuations. In all clinical cases, the dose threshold applied was 50% of the maximum dose and only voxels inside the patient’s body region were considered. For simulations in phantoms the dose threshold applied was 20% of the maximum dose.

#### Photon transport in a MLC

**Fig. 1:**
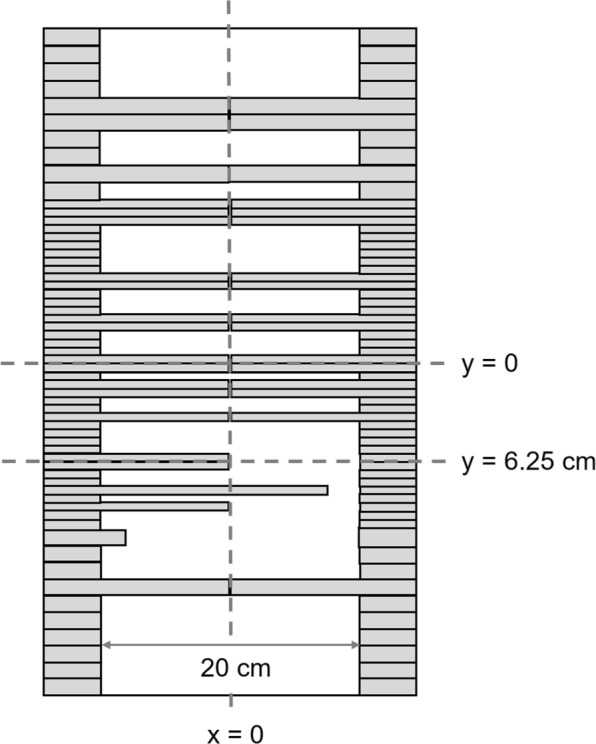
Leaf pattern used to verify the transport through the MLC. Dose profiles were taken in the water phantom along the dashed lines

Dose distributions produced by a 6 MV photon beam were estimated with *p*DPM and PENELOPE. The Varian Millennium 120 MLC was configured with the leaf pattern represented in Fig. 1. This pattern, the same used by Heath and coworkers [25], was chosen because it can assess the effect on the dose of several critical regions of the MLC in a single simulation. The dose distributions were tallied in a water phantom of 40×40×30 cm^3^ with a bin size of 0.2×0.2×0.5 cm^3^. The field size was set to 30×40 cm^2^. A total of 10^9^ histories were simulated to obtain an average standard statistical uncertainty of 0.2%. The evaluation was made by gamma analysis and also by comparing dose profiles taken along critical regions.

#### Photon transport in a multi-layer phantom

Dose distributions produced by a 6 MV photon beam were estimated in a slab phantom consisting of seven 5-cm-thick layers. The phantom dimensions were 40×40×35 cm^3^ with a bin size of 0.5×0.5×0.25 cm^3^. An open field of 10×10 cm^2^ with a SSD = 100 cm was used. The layer materials were (starting from the upstream phantom surface): muscle skeletal (*ρ*=1.04 g/cm^3^), air, lung (*ρ*=0.3 g/cm^3^), muscle skeletal, compact bone (*ρ*=1.85 g/cm^3^), lung and muscle skeletal [26].

#### Simulation of photon beams in clinical cases

Three volumetric-modulated arc therapy (VMAT) clinical cases of head and neck, brain and lung were considered in this work. The head and neck plan consisted of two coplanar hemi-arcs, covering from 0 to 179 degrees. Each arc had 96 control points. Two PTVs were delineated in the left side of the patient neck (see Fig. 4). The prescribed dose were 40 Gy and 44 Gy in 20 fractions to PTV_1_ and PTV_2_, respectively. Two OARs were selected for dose comparison, the left parotid gland and the spinal cord. The lung plan also had two hemi-arcs, from 181 to 0 degrees with 96 control points each. The PTV was a relatively small region with a volume of 6.9 cm^3^ located in the posterior lung wall near the diaphragm. The prescribed dose to that PTV was 52 Gy in 8 fractions. The brain case is a post surgery irradiation of a brain tumor. Two PTV regions were delimited PTV_1_ and PTV_2_ with prescribed doses of 50 Gy and 60 Gy in 25 fractions, respectively. The plan consisted of two coplanar full arcs with 177 control points each. The brain stem OAR was selected for dose comparison. Additionally, a prostate IMRT plan consisting of five fields distributed at angles of 255, 315, 45, 105 and 180 degrees was included in this study. The total number of control points was 621. The prescribed dose to the prostate PTV was 76 Gy in 39 fractions. The bladder and rectum OARs were selected for dose comparison.

The voxelized geometry generated by PRIMO uses the voxel size provided in the CT scan. However, PRIMO allows to set a fixed spatial resolution of the simulation geometry of 0.25 cm^3^. This is done by averaging HU in neighbor voxels, each weighted by the fraction of the volume included in the destination voxel. At the end of the simulation the original CT resolution is recovered by interpolating the dose obtained for the coarser voxel size.

Dose distributions were obtained with *p*DPM, both using the original voxel size and the coarse option, and with PENELOPE only using the original size. The dose distribution obtained with the original CT resolution was used for comparison with PENELOPE. Gamma analysis was applied to all voxels inside the body region.

#### Simulation times

Simulation times obtained with *p*DPM were reported in a previous work [20]. However, that article considered only voxelized geometries. For the present study all simulations were carried out in two Xeon E5-2670V3 CPUs with 12 cores each, and hyper-threading. The compiler used was Intel Fortran v16 for Windows with compilation options /O2 /Qipo /QxP for PENELOPE and /Qopenmp for *p*DPM. PENELOPE is a serial code, hence, simulations were carried out by simultaneously running 32 instances of the code (each one with different initial random number seeds) and letting the operating system (Windows Server 2016) deal with the task assignment to the CPU cores. In order to provide a source of particles for each PENELOPE instance, the source phase-space file must be partitioned prior to starting the simulation. For the phase space used in this work this partitioning process took approximately 15 min. This time was not taken into account in the benchmark. Conversely, *p*DPM genuinely runs in parallel, hence, partitioning of the phase-space file is not necessary. The simulations with *p*DPM used 32 threads. In all cases the simulation time reported corresponds to that required to reach an average standard statistical uncertainty of 1%. The reported dose statistical uncertainties are computed using voxels that score more than 50% of the maximum dose.

## Results

### Photon transport in a MLC

**Fig. 2:**
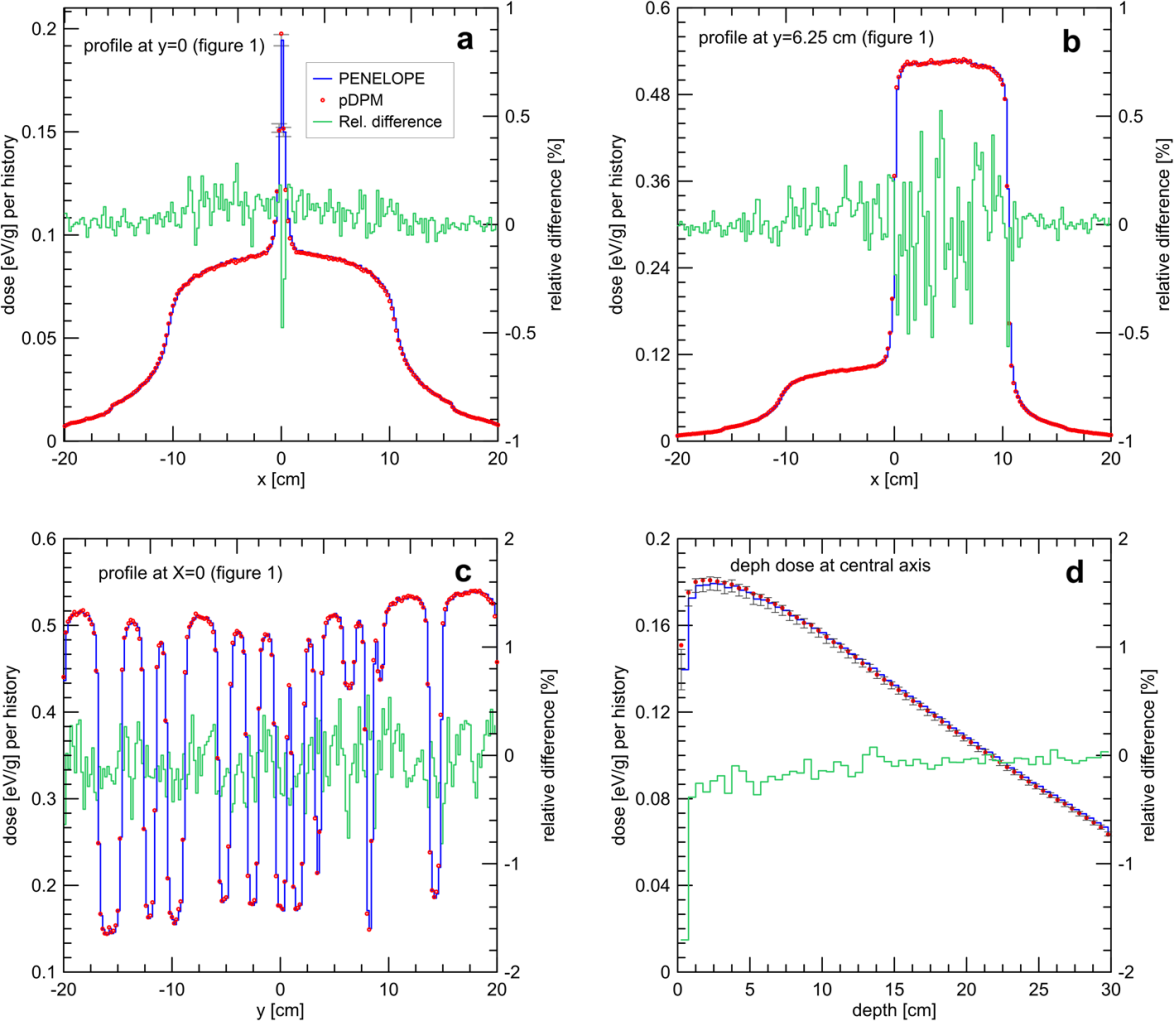
Dose profiles produced with simulations employing *p*DPM and PENELOPE of a 6 MV photon beam and the MLC configured according to the pattern in figure 1. The profiles were taken along critical dose regions. Dose uncertainties are plotted only when they are larger than symbols. Dose differences PENELOPE −*p*DPM relative to the PENELOPE maximum dose are shown in green

A good agreement between the dose distributions obtained with PENELOPE and *p*DPM was obtained for this test. The percentage of points passing gamma analysis with criteria of 1%, 1 mm was 99.5%. Systematic deviations between both dose distributions are small as depicted in Table 1. The good agreement between both distributions can also be observed in the dose profiles shown in Fig. 2. The dose profiles in Fig. 2a were taken in the direction of the *x*-axis at *y*=0 at a depth of 5 cm. From Fig. 1 it can be observed that the dose in this region is mainly produced by radiation traversing the tongue and groove region of the two central leaves. The peak at the center of the profile is produced by radiation traversing the gap between the two opposed rounded leaf tips. Figure 2b represents profiles taken along the *x*-axis direction at off-axis *y*=6.25 cm and 5 cm of depth. They correspond to the transition from the tongue and groove region to an open field, including the effect of the leaf tips. Figure 2c represents profiles taken along the *y*-axis at 5 cm of depth and *x*=0. Figure 2d are depth dose curves taken at the central axis, with a main contribution from radiation traversing the gap between the tips of the central leaves. In all profiles the dose difference between PENELOPE and *p*DPM is lower than 1% of the PENELOPE maximum dose except for the first 0.5 cm of the build-up region where the statistical uncertainty is too large to say. The larger statistical uncertainty in the build-up is due to the presence of contaminant electrons in the beam.

**Table 1:** Systematic differences between the dose distributions estimated with PENELOPE and *p*DPM for the photon test cases included in this work

Test case	*α* [%]	*Δ*[*%*]	*α*[*%*]	*Δ*[*%*]
Described in section *Photon transport in a multi-layer phantom*				
(all voxels)	14.2	-5.0	17.0	0.2
(in the air layer)	97.0	-5.3	0	0
(excluding the air layer)	32.1	-0.2	20.4	0.2
Described in “Photon transport in a MLC” section	26.0	-0.4	13.3	0.3
Head&Neck	32.4	-0.8	17.8	0.7
Lung	36.6	-0.8	11.7	0.5
Brain	30.5	-0.6	7.0	0.7
Prostate	28.1	-0.4	18.2	0.4

They are expressed as the percentage of voxels *α* with a systematic deviation *Δ* given in percentage of the maximum dose

### Photon transport in a multi-layer phantom

**Fig. 3:**
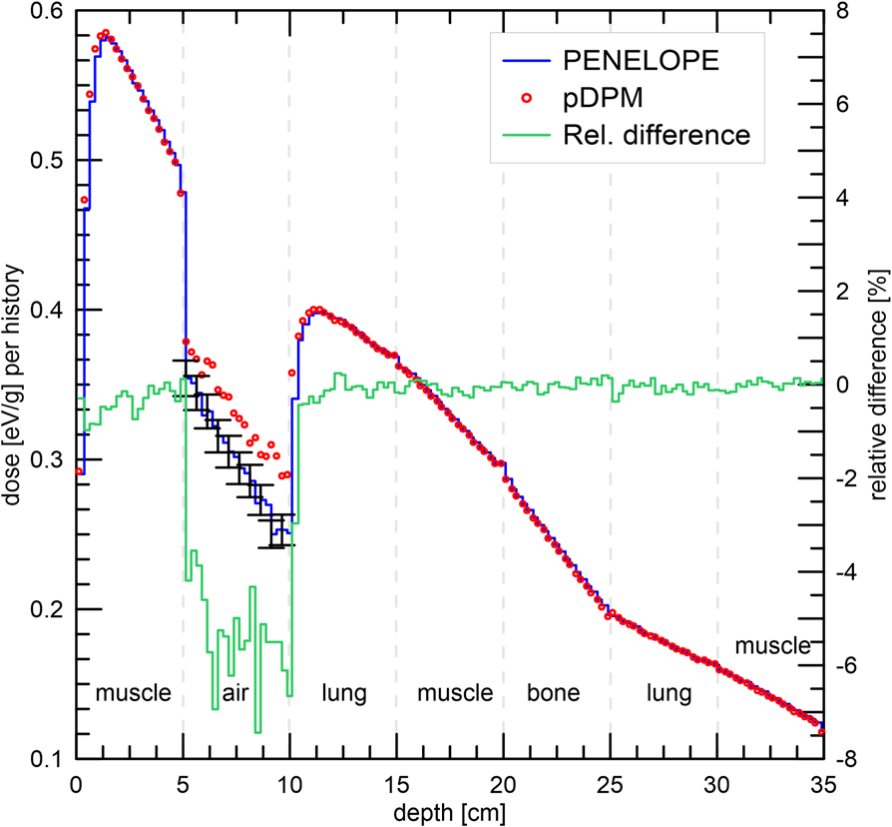
Depth dose curve for PENELOPE and *p*DPM at central axis of a multi-material slab phantom. Dose uncertainties are plotted only for the air, for the rest of materials they are smaller than symbols. Dose differences PENELOPE −*p*DPM relative to the PENELOPE maximum dose are shown in green

The depth dose curve at the central axis of the phantom is shown in Fig. 3. Uncertainties are only shown in the region filled with air. In that region the average standard uncertainty is 1.7*%*. In the remaining regions it is 0.3*%*. Good agreement between the profile obtained with *p*DPM and PENELOPE is observed except for the region filled with air. The agreement between both profiles is better than 1% except for air, where the maximum difference is 24%. From Table 1 it can be seen that systematic differences in the region filled with air range between 5−6*%*.

### Simulation of photon beams in clinical cases

**Fig. 4:**
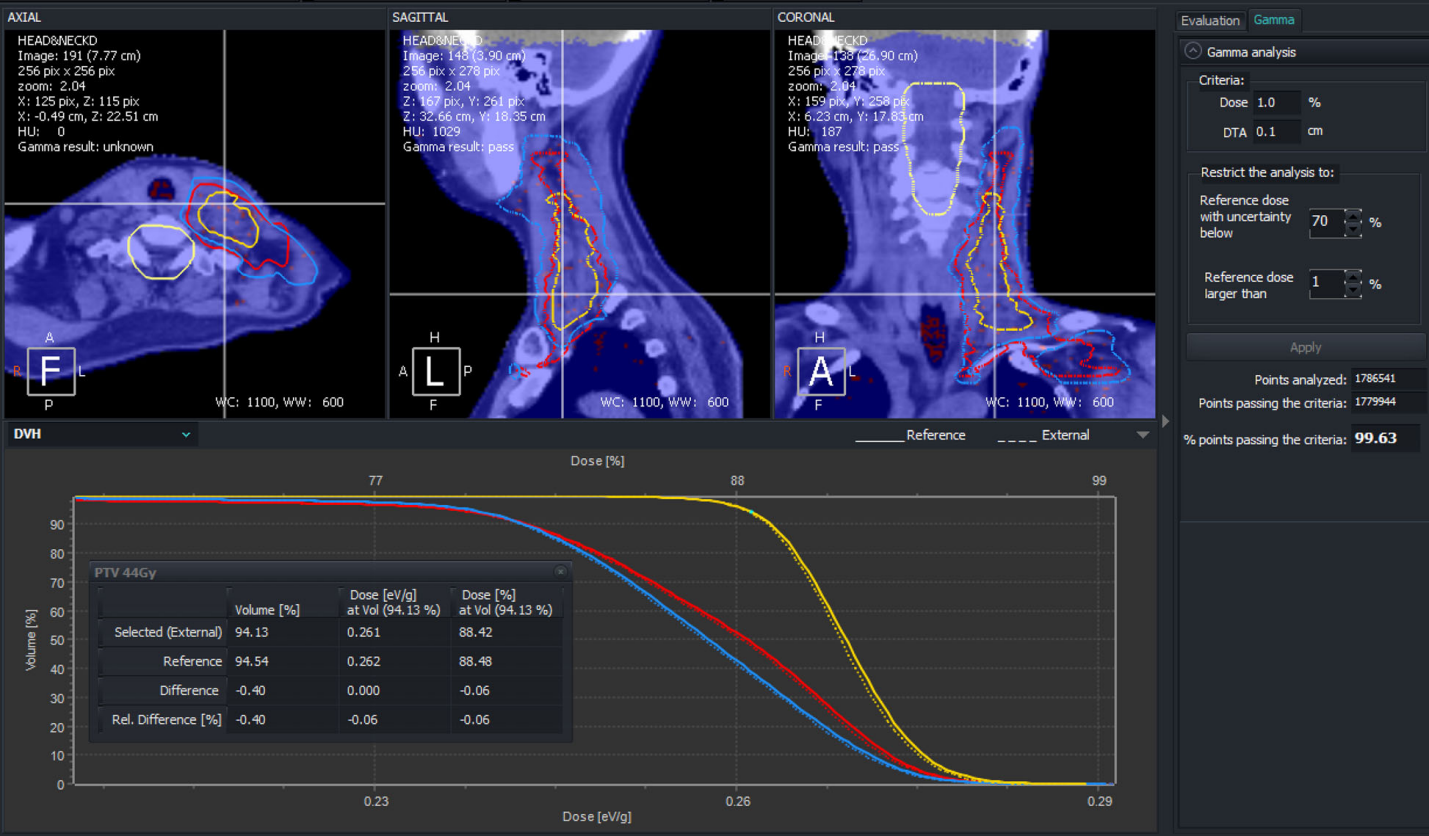
PRIMO screenshot showing the results of 3-D gamma analysis, performed with criteria 1%, 1 mm, for the head and neck case in which PENELOPE and *p*DPM simulations are compared. An excellent agreement, of 99.6%, between both simulations is obtained. The dose-volume histograms of the PTVs, whose contours appear in the upper panels, have been magnified to better expose the small differences between *p*DPM (solid lines) and PENELOPE (dashed lines)

Combined standard uncertainties obtained for the simulations of clinical cases with PENELOPE and *p*DPM were 0.60, 0.77, 0.63 and 0.7 for brain, head and neck, lung and prostate, respectively. In all cases, a good match between both dose distributions was obtained. The fraction of points passing the 3-D gamma analysis inside the body region with criteria of 1%, 1 mm (*Γ*_1,1_) were 99.7%, 99.6%, 99.8% and 99.6%, for the cases of brain, head and neck, lung, and prostate, respectively. Table 2 shows gamma pass rates *Γ*_1,1_ and *Γ*_2,1_ for PTVs and selected OARs. A good agreement was obtained in all cases except for *Γ*_1,1_ of the head and neck PTV_2_ probably due to its small volume (50 cm^3^) and the fact that 1% dose difference is in the range of the average dose uncertainty. However, when the dose difference criterion is set to 2%, gamma pass rate is 100% for that PTV. Figure 4 shows a PRIMO screenshot with the comparison for the head and neck case. Systematic differences were small, within ±0.8*%* of the maximum dose for all cases.

**Table 2:** Fraction of points passing gamma analysis with criteria 1*%*,1 mm (*Γ*_1,1_) and 2*%*,1 mm (*Γ*_2,1_) in the region delimited by the body contour, the PTVs and the OARs

Region	*Γ*_1,1_ [%]	*Γ*_2,1_ [%]
Prostate		
Body	99.8	100
PTV	99.6	100
Rectum	99.7	100
Bladder	100	100
H&N		
Body	99.6	100
PTV_1_	98.0	100
PTV_2_	96.2	100
Spine	100	100
Left Parotid	99.2	99.9
Brain		
Body	99.7	100
PTV_1_	99.4	100
PTV_2_	99.1	100
Brain stem	99.6	100
Lung		
Body	99.6	100
PTV	99.2	100

### Simulation times

Results of the performance benchmark for mixed geometries are shown in Table 3. It can be observed that the speedup of *p*DPM with respect to PENELOPE is moderate. The *p*DPM computational speed is hampered by the fact that the transport through the linac uses the PENELOPE geometry model. Furthermore, the time employed in updating the quadric geometry in dynamic plans is roughly 0.4 s per control point. A more favorable simulation time is obtained when the “coarse” option is used in *p*DPM, as it is shown in the “coarse voxel” column.

**Table 3:** Simulation times in minutes for PENELOPE and *p*DPM to obtain a dose distribution with 1% standard statistical uncertainty for some single field cases and dynamic treatments

		Simulation time [min]			Speedup	
			*p*DPM			
Test case	Voxel size [cm^3^]	penelope	Original voxel	Coarse voxel	Original voxel	Coarse voxel
Described in “Photon transport in a multi-layer phantom” section	0.5×0.5×0.25	37	9.5	-	3.9×	-
Described in “Photon transport in a MLC” section	0.2×0.2×0.5	324	129	-	2.5×	-
Head&Neck VMAT, 194 CP	0.19×0.15×0.19	1061	140	42	7.6×	25.3×
Lung VMAT, 194 CP	0.19×0.14×0.19	331	28	14	11.8×	23.6×
Brain VMAT, 354 CP	0.11×0.2×0.11	687	117	34	5.8×	20.2×
Prostate IMRT, 621 CP	0.18×0.25×0.18	472	64	45	7.3×	10.5×

Clinical cases were simulated with the same voxel size of the original CT scan (original voxel), for both penelope and *p*DPM. Simulation times with *p*DPM for the clinical cases in which the coarse voxel size of (0.25 cm)^3^ was employed are reported in the corresponding column. The speedups of *p*DPM with respect to penelope for the simulation with the original voxel size are given in the *Original voxel* column. The speedup obtained with the coarse option of *p*DPM is also reported. CP stands for control point

## Discussion and conclusions

DPM has been incorporated as an efficient Monte Carlo engine for photon dose estimation in PRIMO since version 0.3.1.1600. It allows the joined simulation of the patient-dependent part of the linac and the patient geometry, thus facilitating dose estimation of dynamic treatments. The version of PRIMO used for this article has been 0.3.1.1681.

PENELOPE and DPM use different physics models. Generally speaking, DPM cross section models are simpler albeit accurate enough for the dynamical range for which the code was designed, that is, low *Z* materials and high energies. In this work, however, we have used *p*DPM to simulate the transport in some of the tungsten elements of the linac head. Despite this fact, the comparisons between PENELOPE and *p*DPM made in this work have not shown a substantial impact on the dose accuracy of DPM physics models simplifications. Thus, a good agreement between the results obtained with PENELOPE and *p*DPM was obtained for the studied clinical cases, in which 99.9% or more of points passed the 3-D gamma analysis with criteria 2%, 1 mm and systematic differences were within ±0.8*%* of the maximum dose. The discrepancy observed in the multi-layer phantom, related to the transport in air, is due to an artifact of the cross section interpolation algorithm for low energy electrons in air. The dose is not biased in any other material, nor at the interfaces with air. Investigations to correct this artifact are currently in progress.

The speedup factor obtained with *p*DPM with respect to PENELOPE was in all clinical cases between 6 and 12. This speedup factor is further increased when voxels are grouped using the “coarse” option, attaining values in the order of 20. These factors are reached although the transport in the linac geometry hinders the overall efficiency of *p*DPM owing to the use of the PENELOPE geometry model.

## Abbreviations

CT: Computerized tomography
DTA: Distance to agreement
GPU: Graphics processing unit
OAR: Organ-at-risk
PSF: Phase-space file
PTV: Planning target volume
VMAT: Volumetric-modulated arc therapy
